# Supplementary material for: Enhancing Advance Directive Completion Among Older Adults in the Geriatrics Clinic in Indiana, USA: A Quality Improvement Initiative
Source: Healthcare (Basel). 2025 Nov 27;13(23):3086. doi: 10.3390/healthcare13233086 (PMC12692473; doi:10.3390/healthcare13233086)
Supplement: Supplementary file 1 [file healthcare-13-03086-s001.zip › healthcare-3908392-supplementary.pdf]

**SQUIRE 2.0 Item**	**Addressed in Manuscript**	**Section**
Title	Identifies focus and design (Quality Improvement on Advance Directives)	Title
Abstract	Structured with Background, Objective, Methods, Results, Conclusions	Abstract
Problem Description	Describes low AD completion and end-of-life care gap	Introduction
Available Knowledge	Reviews existing ACP literature and disparities	Introduction
Rationale	Explains focus on ADs and national average goal	Introduction
Specific Aims / Study Question	Increase AD completion to ≥46% among older adults	Introduction
Context	IUHPC geriatrics clinic, consistent staff, timeframe	Methods
Intervention	PDSA workflow, roles, scripts, materials	Methods
Study of the Intervention	Data capture, feedback loops	Methods
Measures	Defined primary outcome (AD completion proportion)	Methods
Analysis	Statistical tests, CI, logistic regression	Methods
Results	Descriptive and aggregate pre/post analyses	Results
Interpretation	Discusses implications, barriers, equity	Discussion
Limitations	Small N, single site, documentation bias	Discussion
Conclusions	Summarizes findings and sustainability	Conclusions
Ethical Considerations	IRB waiver, de-identification	Methods
Funding / Conflicts	Declared none	End matter

Supplementary Tables and Figures

Figure S1. AD Completion Rates by Month at IUHPC Geriatrics Clinic (April-August [illegible]

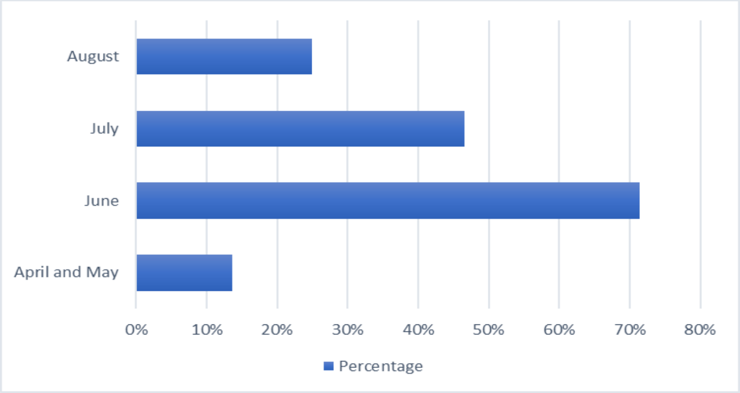

Table S1. AD Completion Rates by Month at IUHPC Geriatrics Clinic (April–August 2024).

Months	Completed ADs	Total Patients	Percentage
**April - May**	3	22	13.6%
**June**	10	14	71.4%
**July**	7	15	46.6%
**August**	4	16	25%
**Total**	24	67	35.82%

Figure S2. AD Completion Rates by Month at IUPHC Geriatrics Clinic (September-November 2024).

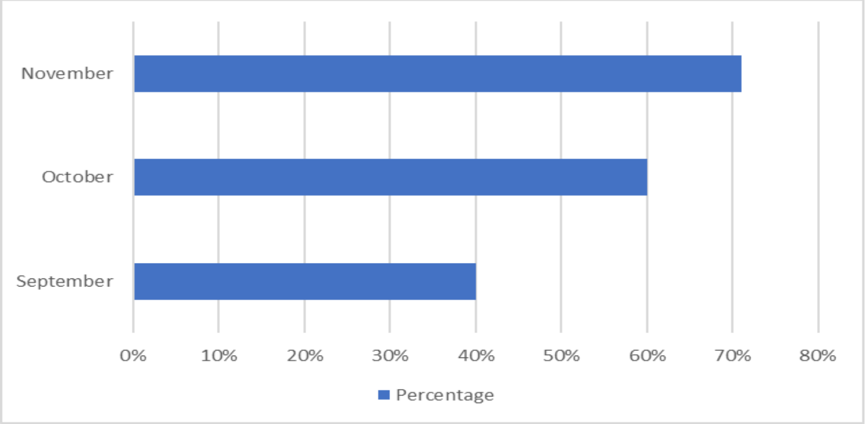

Table S2. AD Completion Rates by Month at IUPHC Geriatrics Clinic (September–November 2024).

Months	Completed ADs	Total Patients	Percentage
**September**	6	15	40%
**October**	9	15	60%
**November**	10	14	71.1%
**Total**	25	44	56.8%

Figure S3. AD Completion Rates by Month at IUPHC Geriatric Clinic (January-June 2025).

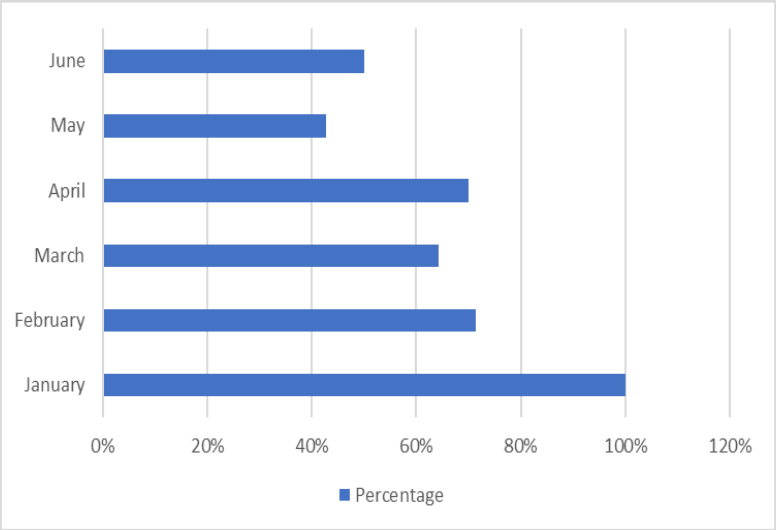

Table S3. AD Completion Rates by Month at IUPHC Geriatrics Clinic (January–June 2025).

Months	Completed ADs	Total Patients	Percentage
**January**	2	2	100%
**February**	5	7	71.4%
**March**	9	14	64.2%
**April**	7	10	70%
**May**	6	14	42.8%
**June**	4	8	50%
**Total**	33	55	66.4%

INDIANA
ADVANCE CARE DIRECTIVE

INDIANA HEALTH CARE REPRESENTATIVE:
A Health Care Representative is a person chosen by you to make healthcare decisions, including end-of- life decisions, if you are unable to make your own. It is a good idea to talk with this person about your preferences ahead of time. A doctor will determine if you are unable to make your own decisions.

My name (also known as "Declarant"):

______________________________ ____________________

Full Legal Name | Date of Birth (MM/DD/YYYY)

My Health Care Representative can make decisions for me if I cannot make and share my own health care decisions. My Health Care Representative must follow my wishes and values. My values include my ideas about dignity and quality of life. If my Health Care Representative does not know my wishes, my Health Care Representative must act in good faith and make decisions in my best interests. These decisions include but are not limited to:

- Agreeing to medical treatment
- Refusing medical treatment
- Stopping medical treatment
- Arranging comfort care

I want the following person to be my Health Care Representative (HCR):

______________________________ ____________________

HCR Name | HCR Phone Number

If my primary HCR named above is not able or available to act for me, I want the following person to be my backup Health Care Representative:

______________________________ ____________________

Backup HCR Name | Backup HCR Phone Number

OPTIONAL STATEMENT OF PREFERENCES:
I would like to provide some additional guidance for my Health Care Representative on my end-of-life preferences. (Please select only one option below).

☐ The ***quality of my life*** is more important than the length of my life. If I am unable to make my own decisions and my attending physician believes that I will not recover, I do not want treatments to prolong my life or delay my death. Instead, I would want treatment or care to make me comfortable and to relieve me of pain.

☐ ***Staying alive*** is more important to me, no matter how sick I am or how unlikely my chances for recovery are. I want my life to be prolonged to the greatest extent possible, in accordance with reasonable medical standards.

☐ I choose to NOT complete this section at this time.

REQUIRED SIGNATURES:
By signing this form, I cancel and revoke every health care power of attorney I signed in the past.

_________________________________ _________________________
Signature (Declarant) Date

____
Printed Name (Declarant)

This form must be either signed by 2 adult witnesses (below left) or notarized (below right) to be legally valid.

SIGNATURE OF 2 ADULT WITNESSES

Each of the undersigned Witnesses confirms that he or she has received satisfactory proof of the identity of the Declarant and is satisfied that the Declarant is of sound mind and has the capacity to sign the above Advance Directive. **At least one of the undersigned Witnesses is not a spouse or other relative of the Declarant.**

____
Signature of Adult Witness 1

____
Printed Name of Adult Witness 1

____
Date

____
Signature of Adult Witness 2

____
Printed Name of Adult Witness 2

____
Date

________ Initial here if the Witnesses participated by phone.

NOTARIZATION

STATE OF INDIANA)
) SS:
COUNTY OF __________)

Before me, a Notary Public, personally appeared _____________________ [*name of signing Declarant*], who acknowledged the execution of the foregoing Advance Directive as his or her voluntary act, and who, having been duly sworn, stated that any representations therein are true.

Witness my hand and Notarial Seal on this _____ day of ____________, 20___.

____
Signature of Notary Public

____
Notary's Printed Name *(if not on seal)*

____
Commission Number *(if not on seal)*

____
Commission Expires *(if not on seal)*

____
Notary's County of Residence

INDIANA PHYSICIAN ORDERS FOR SCOPE OF TREATMENT (POST)

State Form 55317 (R / 11-16)
Indiana State Department of Health – IC 16-36-6

INSTRUCTIONS: This form is a physician's order for scope of treatment based on the patient's current medical condition and preferences. The POST should be reviewed whenever the patient's condition changes. A POST form is voluntary. A patient is not required to complete a POST form. A patient with capacity or their legal representative may void a POST form at any time by communicating that intent to the health care provider. Any section not completed does not invalidate the form and implies full treatment for that section. HIPAA permits disclosure to health care professionals as necessary for treatment. The original form is personal property of the patient. A facsimile, paper, or electronic copy of this form is a valid form.

Patient Last Name	Patient First Name	Middle Initial

Birth Date *(mm/dd/yyyy)*	Medical Record Number	Date Prepared *(mm/dd/yyyy)*

	**DESIGNATION OF PATIENT'S PREFERENCES:** The following sections (A through D) are the patient's current preferences for scope of treatment.
**A** *Check One*	**CARDIOPULMONARY RESUSCITATION (CPR):** Patient has no pulse AND is not breathing<br>☐ Attempt Resuscitation/CPR ☐ Do Not Attempt Resuscitation/DNR<br>☐ When not in cardiopulmonary arrest, follow orders in **B, C** and **D**
**B** *Check One*	**MEDICAL INTERVENTIONS:** If patient has pulse AND is breathing OR has pulse and is NOT breathing<br>☐ <u>Comfort Measures (Allow Natural Death)</u>: Treatment Goal: Maximize comfort through symptom management. Relieve pain and suffering through the use of any medication by any route, positioning, wound care and other measures. Use oxygen, suction and manual treatment of airway obstruction as needed for comfort. Patient prefers no transfer to hospital for life-sustaining treatments. Transfer to hospital only if comfort needs cannot be met in current location.<br>☐ <u>Limited Additional Interventions</u>: Treatment Goal: Stabilization of medical condition. In addition to care described in Comfort Measures above, use medical treatment for stabilization, IV fluids (hydration) and cardiac monitor as indicated to stabilize medical condition. May use basic airway management techniques and non-invasive positive-airway pressure. Do not intubate. Transfer to hospital if indicated to manage medical needs or comfort. Avoid intensive care if possible.<br>☐ <u>Full Intervention</u>: Treatment Goal: Full interventions including life support measures in the intensive care unit. In addition to care described in Comfort Measures and Limited Additional Interventions above, use intubation, advanced airway interventions, and mechanical ventilation as indicated. Transfer to hospital and/or intensive care unit if indicated to meet medical needs.
**C** *Check One*	**ANTIBIOTICS:**<br>☐ Use antibiotics for infection only if comfort cannot be achieved fully through other means.<br>☐ Use antibiotics consistent with treatment goals.
**D** *Check One*	**ARTIFICIALLY ADMINISTERED NUTRITION:** Always offer food and fluid by mouth if feasible.<br>☐ No artificial nutrition.<br>☐ Defined trial period of artificial nutrition by tube. (Length of trial: ________ Goal: ______________________)<br>☐ Long-term artificial nutrition.
	**OPTIONAL ADDITIONAL ORDERS:**
	**SIGNATURE PAGE:** This form consists of two (2) pages. Both pages must be present. The following page includes signatures required for the POST form to be effective.

Patient Name:__________________________ Date of Birth *(mm/dd/yyyy)*:__________________________

SIGNATURE OF PATIENT, LEGAL REPRESENTATIVE, OR PROXY: In order for the POST form to be effective, the patient, legal representative, or proxy must sign and date the form below.

E

SIGNATURE OF PATIENT, LEGAL REPRESENTATIVE, OR PROXY
My signature below indicates that the physician, advanced practice registered nurse, or physician assistant (or their designee) discussed with me the above orders and the selected orders correctly represent the decisions made during this discussion.

Signature *(required)*	Print Name *(required)*	Date *(mm/dd/yyyy)* *(required)*

F

CONTACT INFORMATION FOR LEGAL REPRESENTATIVE OR PROXY IN SECTION E *(IF APPLICABLE)*: If the signature above is other than patient's, add contact information for the representative or proxy.

Relationship of representative or proxy identified in Section E if patient does not have capacity	Address *(number and street, city, state, and ZIP code)*	Telephone Number

PHYSICIAN ORDER:

A POST form may be executed only by an individual's treating physician, advanced practice registered nurse, or physician assistant, and only if:

(1) the treating physician, advanced practice registered nurse, or physician assistant has determined that:
- (A) the individual is a qualified person; and
- (B) the medical orders contained in the individual's POST form are reasonable and medically appropriate for the individual; and

(2) the qualified person, representative, or proxy has signed and dated the POST form

A qualified person is an individual who has at least one (1) of the following:

(1) An advanced chronic progressive illness.

(2) An advanced chronic progressive frailty.

(3) A condition caused by injury, disease, or illness from which, to a reasonable degree of medical certainty:
- (A) there can be no recovery; and
- (B) death will occur from the condition within a short period without the provision of life prolonging procedures.

(4) A medical condition that, if the person were to suffer cardiac or pulmonary failure, resuscitation would be unsuccessful or within a short period the person would experience repeated cardiac or pulmonary failure resulting in death.

G

DOCUMENTATION OF DISCUSSION: Orders discussed with *(check one)*:

☐ Patient (patient has capacity) ☐ Health Care Representative ☐ Legal Guardian
☐ Parent of Minor ☐ Health Care Power of Attorney ☐ Proxy

H

SIGNATURE OF TREATING PHYSICIAN / ADVANCED PRACTICE REGISTERED NURSE / PHYSICIAN ASSISTANT
My signature below indicates that I or my designee have discussed with the patient, patient's representative, or proxy the patient's goals and treatment options available to the patient based on the patient's health. My signature below indicates to the best of my knowledge that these orders are consistent with the patient's current medical condition and preferences.

Signature of Treating Physician / APRN / PA *(required)*	Print Treating Physician / APRN / PA Name *(required)*	Date *(mm/dd/yyyy)* *(required)*
Physician / APRN / PA office telephone number	Physician / APRN / PA License Number	Health Care Professional preparing form if other than the physician / APRN / PA

I

APPOINTMENT OF HEALTH CARE REPRESENTATIVE: As a patient you have the option to appoint a representative to serve as your health care representative pursuant to IC 16-36-7. You are not required to designate a health care representative for this POST form to be effective. You are encouraged to consult with your attorney or other qualified individual about advance directives that are available to you. Forms and additional information about advance directives may be found on the IDOH web site at https://www.in.gov/health/cshcr/indiana-health-care-quality-resource-center/advance-directives-resource-center/ .
